# Host Restriction Factors APOBEC3G/3F and Other Interferon-Related Gene Expressions Affect Early HIV-1 Infection in Northern Pig-Tailed Macaque (*Macaca leonina*)

**DOI:** 10.3389/fimmu.2018.01965

**Published:** 2018-08-28

**Authors:** Wei Pang, Jia-Hao Song, Ying Lu, Xiao-Liang Zhang, Hong-Yi Zheng, Jin Jiang, Yong-Tang Zheng

**Affiliations:** ^1^Key Laboratory of Animal Models and Human Disease Mechanisms of the Chinese Academy of Sciences/Key Laboratory of Bioactive Peptides of Yunnan Province, Kunming Institute of Zoology, Chinese Academy of Sciences, Kunming, China; ^2^Institute of Health Sciences, Anhui University, Hefei, China; ^3^Faculty of Life Science and Technology, Kunming University of Science and Technology, Kunming, China; ^4^Kunming Primate Research Center of the Chinese Academy of Sciences, Kunming Institute of Zoology, Chinese Academy of Sciences, Kunming, China

**Keywords:** northern pig-tailed macaques, HIV-1_NL4-R3A_, stHIV-1sv, neutralizing antibodies, IFN-I signaling, APOBEC3

## Abstract

The northern pig-tailed macaques (NPMs) lack TRIM5α, an antiviral restriction factor, and instead have TRIM5-CypA. In our previous study, we demonstrated that HIV-1_NL4−3_ successfully infected NPMs and formed a long-term viral reservoir *in vivo*. However, the HIV-1-infected NPMs showed relatively high viremia in the first 6 weeks of infection, which declined thereafter suggesting that HIV-1 _NL4−3_ infection in these animals was only partly permissive. To optimize HIV-1 infection in NPMs therefore, we generated HIV-1_NL4−R3A_ and stHIV-1sv, and infected NPMs with these viruses. HIV-1_NL4−R3A_ and stHIV-1sv can replicate persistently in NPMs during 41 weeks of acute infection stage. Compared to the HIV-1_NL4−R3A_, stHIV-1sv showed a notably higher level of replication, and the NPMs infected with the latter induced a more robust neutralizing antibody but a weaker cellular immune response. In addition, IFN-I signaling was significantly up-regulated with the viral replication, and was higher in the stHIV-1sv infected macaques. Consequently, the sequences of pro-viral *env* showed fewer G-A hyper-mutations in stHIV-1sv, suggesting that *vif* gene of SIV could antagonize the antiviral effects of APOBEC3 proteins in NPMs. Taken together, NPMs infected with HIV-1_NL4−R3A_ and stHIV-1sv show distinct virological and immunological features. Furthermore, interferon-related gene expression might play a role in controlling primary HIV-1_NL4−R3A_ and stHIV-1sv replication in NPMs. This result suggests NPM is a potential HIV/AIDS animal model.

## Introduction

Non-human primates (NHPs) are the preferred experimental animals for HIV-1/AIDS research. Previous studies have reported that most macaques can be infected by SIV, of which the rhesus macaque (*Macaca mulatta*), cynomolgus macaque (*Macaca fascularis*) and pig-tailed macaque (*Macaca nemestrina*) are widely used as the animal models of AIDS (1). However, due to the genetic differences between SIV and HIV-1, these models have limitations in HIV/AIDS research (2). Therefore, the development of NHP models that can be challenged with HIV-1 would undoubtedly facilitate the evaluation of AIDS prevention and treatment strategies. Pig-tailed macaques can be infected with HIV-1, as they lack the HIV-1 blocking protein TRIM5α (3–7). Based on their geographical distribution and morphology differences, pig-tailed macaques are divided into three species: sunda pig-tailed macaque (*M. nemestrina*), northern pig-tailed macaque (*M. leonina*), and mentawai macaque (*M. pagensis*) (8). The sunda pig-tailed macaques (SPMs) are the most frequently used species for HIV-1 infection (9–18).

The northern pig-tailed macaques (NPMs) are found widely in China and the Southeast Asian countries (19). Prior to this study, we found that the NPM lacks the HIV-1 blocking protein TRIM5α, and instead has TRIM5-CypA (3, 20), which makes it susceptible to HIV-1 infection. In addition, PBMCs isolated from the pig-tailed macaques can be infected with HIV-1, more robustly with the stHIV-1sv (21). We also found that NPMs could be successfully infected by the HIV-1_NL4.3_ strain, resulting in relatively high viremia for 6 weeks. Although the viral load in the blood dropped quickly thereafter, cell-associated HIV-1 DNA and RNA persisted in the peripheral blood and lymphoid organs for about 3 years. Furthermore, replication-competent HIV-1 could be successfully reactivated both *ex vivo* and *in vivo* in response to co-stimulation with the latency-reversing agents JQ1 and prostratin. These results suggest HIV-1 can replicate at a low level and form a long term viral reservoir in NPMs (22), making them a potential animal model for HIV/AIDS research.

Previous studies have demonstrated that host restriction factors other than TRIM5α, such as APOBEC3G and APOBEC3F (23, 24), can strongly inhibit HIV-1 infection upon induction by type I interferons. Following HIV-1 infection, some cells can produce type I interferons, which induce the expression of IFN-stimulated genes (ISGs) via the JAK-STAT signal pathway (25). At the same time, interferon signaling and regulating genes are also induced, which may inhibit virus replication. HIV-1 can antagonize the human forms of APOBEC3 proteins by degrading them through its *vif* protein sequences. However, it fails to antagonize the macaque APOBEC3 proteins, and thus cannot effectively replicate in SPMs (9–18, 26). A genetically engineered stHIV-1 strain which only differs from HIV-1 in harboring the *vif* gene from SIVmac239, can robustly replicate and even result in AIDS like symptoms in the SPMs which were depleted of CD8+ cells before infection (27–30).

SPMs have been well demonstrated progress to AIDS more rapidly than rhesus macaques after SIVmac239 infection (31). However, in our previous studies, we found that NPMs progressed to AIDS much more slowly than rhesus macaques, and maintained superior CD4+ T cell homeostasis during SIVmac239 infection (32, 33). These results implied great distinctions in physiological and immunologic responses, as well as genetic background between SPMs and NPMs.

In this study, to establish an optimal model of NPM that can be infected with HIV-1, we generated two engineered HIV-1 strains: HIV-1_NL4−R3A_ and stHIV-1sv. Both of them originated from HIV-1_NL4.3_ strains: HIV-1_NL4−R3A_ contains HIV-1_R3A_ *env* gene, this virus leads to a rapid disease progress (34), while stHIV-1sv contains a macaque-adapted HIV-1 *env* gene from SHIV_KB9_ and a *vif* gene from SIVmac239 (28), which enables its replication in the PBMCs of NPMs. After inoculating NPMs with the two HIV-1 strains, the plasma viral loads peaked 1-2 weeks post infection (wpi) and persistent in the acute stage. The plasma viral loads were significantly higher in the NPMs infected by stHIV-1sv compared to HIV-1_NL4−R3A_. Peripheral blood CD4+ T-cell counts moderately fluctuated, but did not decrease significantly over a prolonged period of infection. Antibodies, neutralizing antibody and cellular immune responses appeared 4 weeks after infection, after which HIV-1 replication significantly decreased.

To determine the reasons for the low level of HIV-1 _NL4−R3A_ and stHIV-1sv replication in the NMPs during primary infection, the possible anti-viral effects of interferon genes and APOBEC3G/3F were studied. Interferon genes expression peaked at 1–3 weeks after infection before gradually declining to the basal levels, which was consistent with the viral load. The stHIV-1sv *env* gene had less mutations induced by APOBEC3 family, suggesting that the *vif* could better antagonize the antiviral effect of APOBEC3G/3F. As expected, substitution with SIVmac239 *vif* improved the infection model. This result suggests NPM is a potential HIV/AIDS animal model.

## Materials and methods

### HIV-1_NL4-R3A_ and stHIV-1sv strains

The provirus plasmids of HIV-1_NL4−R3A_ and stHIV-1sv were donated by Prof. Liguo Zhang (Institute of Biophysics, Chinese Academy of Sciences) and Guang-Xia Gao (Institute of Biophysics, Chinese Academy of Sciences) respectively. HIV-1_NL4−R3A_ and stHIV-1sv strains were produced in 293T cells (Type Culture Collection, Chinese Academy of Sciences, TCC CAS) by transfecting the provirus plasmids using Lipofectamine^TM^ 2000 according to the manufacturer's instructions (Invitrogen). Viruses were harvested 48 or 72 h post-transfection by centrifuging the media at 3,000 g for 10 min to deposit the cellular debris, and 1 ml aliquots of the virus containing supernatants were frozen at −80°C until use. Viral titers were determined in TZM-bl reporter cells. Briefly, TZM-bl reporter cells were seeded in a 96-well plate, and then infected with serial 5-fold dilutions of the virus stock. After 48 h, the cells were lysed, treated with Bright-Glo^TM^ Reagent, and the relative luminescence units (RLU) were measured in the luminometer (Molecular Devices).

### Animals and infection

Eight northern pig-tailed macaques were obtained from the Kunming Primate Research Center, Kunming Institute of Zoology, Chinese Academy of Sciences. They were housed and fed in accordance with the regulations of the American Association for Assessment and Accreditation of Laboratory Animal Care (AAALAC). All experimental procedures were approved by the Institutional Animal Care and Use Committee of the Kunming Institutional of Zoology, Chinese Academy of Sciences. Macaques used in this study were confirmed to be free from simian immunodeficiency virus (SIV), simian type-D retrovirus, and simian T-lymphotropic virus type-1 by Nested-PCR screening. Four macaques were selected (6–8 years old; female: *n* = 2; male: *n* = 2) for HIV-1_NL4−R3A_ and stHIV-1sv infection respectively. Blood was collected by venipuncture, and PBMCs were isolated by Ficoll density gradient centrifugation. The PBMCs (1 × 10^7^ cells) were infected with HIV-1_NL4−R3A_ or stHIV-1sv particles at the multiplicity of infection (MOI) of 0.01. Three days later, autologous PBMCs plus 10^6^ TCID50 cell free viruses were injected intravenously into each animal.

### Plasma viral load measurement

The plasma viral RNA was extracted by high pure viral RNA Kit (Roche) according to the manufacturer's instructions. The viral load was quantified by a real-time PCR method based on amplification of a HIV-1_NL4.3_-derived Gag coding sequence as described previously (22).

### Quantification of PBMC-associated HIV-1 RNA and DNA

PBMCs (~4 × 10^6^) were split into two equal parts to measure cell-associated HIV-1 DNA and RNA. Total cellular DNA was extracted using the QIAmp DNA Blood Mini Kit (Qiagen, German) and eluted in 100 μl. HIV-1 DNA and RNA was quantified by qPCR as previously reported (22).

### Anti-HIV-1 antibody

HIV-1-specific antibodies in the peripheral blood were measured by MAXI HIV-1 western blot kit (MAXIM) according to the manufacturer's instructions. HIV-1 neutralization assays were performed in TZM-bl cells infected with the HIV-1_NL4−R3A_ or stHIV-1sv. Briefly, 1 × 10^4^ TZM-bl cells were seeded per well of 96-well microtiter plates in DMEM with 10% fetal calf serum (FCS). After 24 h, serial dilutions of heat-inactivated plasma from the infected NPMs were incubated with a fixed inoculums (MOI = 0.01) of HIV-1_NL4−R3A_ or stHIV-1sv for 30 min at 37°C; the pre-infection plasma from each animal were used as the negative controls. The virus/plasma mixtures were then applied to TZM-bl cells and incubated for 48 h. The cells were lysed and the luciferase activity in the cell lysates was determined using the Bright-GloTM Luciferase Assay System (Promega, Madison, USA). The limit of detection of neutralization assay was 40-fold dilution of heat-inactivated plasma.

### Flow cytometry

Monoclonal antibodies (mAbs) and reagents were purchased from BD Biosciences (BD, Franklin Lakes, NJ, USA). Surface staining for lymphocyte immuno-phenotyping was performed as previously described. Briefly, 100 μl of whole blood was lysed with FACS Lysing Buffer for 10 min at room temperature. After washing with DPBS containing 2% newborn calf serum and 0.09% sodium azide (staining buffer), the residual leukocytes were re-suspended in staining buffer containing the relevant mAbs for 30 min on ice, and then fixed with 4% paraformaldehyde in PBS. The stained cells were acquired in a BD FACSVerse cytometer driven by FACSuite software (version 1.0.3; BD), and FlowJo software (version 7.6.1; TreeStar) was used for data analysis. The surface mAbs used were: anti-CD3-PE/-APC-Cy7 (clone SP34-2), anti-CD4-FITC/-PerCP-Cy5.5 (clone L200), and anti-CD8α-PE-Cy7.

### CTL response to HIV-1

HIV-1–specific cytotoxic T lymphocytes (CTL) were detected by a Human IFN-γ ELISPOT kit (Dakawei biotech, ShenZhen, China) according to the manufacturer's instructions. Briefly, 96-well multiscreen plates pre-coated with anti-human interferon-γ were reactivated with RPMI-1640 for 5–10 min at 37°C. After removing the supernatants, 2 × 10^5^ NPM PBMCs were seeded per well in 100 μl RPMI 1640 supplemented with 10% FBS, and incubated with pools of 11 overlapping 15-amino-acid peptides covering the Env, Pol, and Gag proteins of HIV-1 consensus Subtype B. Each peptide was used at a working concentration of 1 μg/ml. Concanavalin A (10 μg/ml) was used as the positive control, and unstimulated cells were used as negative control. After 24 h, wells were imaged using an ELISPOT reader, and spots were counted using an automated program using parameters like size, intensity and gradient. The limit of detection was set at 100 spot-forming cells per million PBMCs.

### Quantitative RT-PCR for IFN related genes

Total RNA was isolated from PBMCs using Trizol reagent and cDNA was generated with PrimeScript^TM^ RT regent Kit with gDNA Eraser. Real-time qPCR reactions were performed on a ViiA7 Real-Time PCR System using SYBR Premix Ex Taq II. The primers for amplifying the IFN related genes are listed in Table 1. Expression levels of target genes were analyzed using the comparative cycle threshold (Ct) method, where Ct is the cycle threshold number normalized to that of the RPL13A mRNA. Fold-change was calculated using the ΔΔCt method by dividing the normalized quantity of post-infection samples by that of the pre-infection samples.

**Table 1:** Primers used to amplify IFN related genes.

**Gene**	**Forward (5^′^-3^′^)**	**Reverse (5^′^-3^′^)**
RPL13A	AAGGTGTTTGACGGCATCCC	CTTCTCCTCCAAGGTGGCTGT
IFNA2	CCTGGCACAAATGAGGAGAAT	GGAACTGGTTGCCAAACTCC
IFNG	GAGTGTGGAGACCATCAAGGA	ACTGCTTTGCGTTGGACATT
IFNK	ATTGCTGGCACCCTATCCCT	TTCTTGGGGCAACTCAAAAGC
IRF7	AGCTGCATGTTCCTGTACG	TCAGCAGTTCCTCCGTGTAG
IRF9	TGAGCCACAGGAAGGTACAG	ACGCCCGTTGTAGATGAAGG
STAT1	TCATCAGCAAGGAGCGAGAG	CGCATGGAAGTAAGGTTCGC
STAT2	GGCTCTCAGTTGGCAGTTCT	CGCTTAGTGAAGTCAGCCCA
ADAR	ATGACCAGCCCGAAGGTATG	AGCTCGCCAATCTTCCTGAC
SOCS1	AACTCGCACCTCCTACCTCT	AAATAAAGCCAGAGACCCTCCC
CXCL10	TGCTGCCTTGTCTTTCTGACT	ATGCTGATGCAGGTACAGCG
IFIT1	AGGAAACACCCACTTCGGTC	CTGCCCTTTTGTAGCCTCCT
IFIT3	GAAGCCGAAGGAGAGCAGTT	CCAATGCCCGTTGAAACAGT
IFI27	ACTCTCCGGGTTGACCAGAT	TGGCACGGTTCTCTTCTCTG
IFI44	TGATAAACGCTGGTGTGGTACA	TGGACTTCCTCTAGCTTGGAC
MX1	AAAGCCCAGAATACCATCGCC	TGTCAGGAGGTTGATTGCCC
MX2	AACTTGGTGGTGGTTCCCTG	GGTGCCCCTGTCCATTAGAT
OAS1	GCAGAAAGAGGGCGAGTTCT	GTGCTTGACTAGGCGGATGA
OAS2	CCTGGAGCTGGTCACACAAT	CCTGGTTTTCTGCAACTGGC
OAS3	TTGAGGCATGTCAACGGGAG	CAGCACGTCAAAGTCCACAC
A3G	CCTCAAATCAGAAACATGGTGG	AACCAGCGGAGGAATCTCATCT
A3F	CTTTAATAACAGACCCATCCTT	GTTGCCACAGAACCGAGA
Tetherin	CTCCTGGTCATAGTGCTTCTGG	CATTGCGACACTCCATCACTG

### Hyper-mutation of viral integrated DNA in NPMS *in vivo*

Total DNA was extracted from PBMCs of HIV-1_NL4−R3A_ or stHIV-1_sv_ infected NPMs by QIAamp DNA minikit (Qiagen). The *env* gene was amplified by nest-PCR (29), using the following primers: HIV-env-13-5′-CCACTCTATTTTGTGCATCAGA-3′; HIV-env-12-5′-CCTGGTGGGTGCTACTCCTA-3′ in round 1, and HIV-env-9-5′-CTACCCGGGCATATGATACAGAGGTACATAATGTCTGGGC-3′; HIV-env-10-5′-CGCTCTAGACACTTCTCCAATTGTCCCTCAT-3′ in round 2. The amplification conditions were same in both rounds: 94°C for 5 min, followed by 35 cycles of 94°C for 30 s, 50°C for 30s, and 72°C for 90 s, and a final extension at 72°C for 10 min. PCR products were purified by Cycle Pure Kit (Sangon Biotech; shanghai, China) and cloned into pMD-19T vector, which was transformed into *E*. *coli* DH5α cells for amplification and sequencing (Sangon Biotech; shanghai, China). Env DNA sequence hypermutation analysis was performed using Hypermut 2.0 (http://www.hiv.lanl.gov/content/sequence- /HYPERMUT/hypermut. html).

## Results

### HIV-1_NL4-r3A_ and stHIV-1sv can replicate more robustly than HIV-1_NL4-3_ in NPM PBMCs

**Figure 1:**
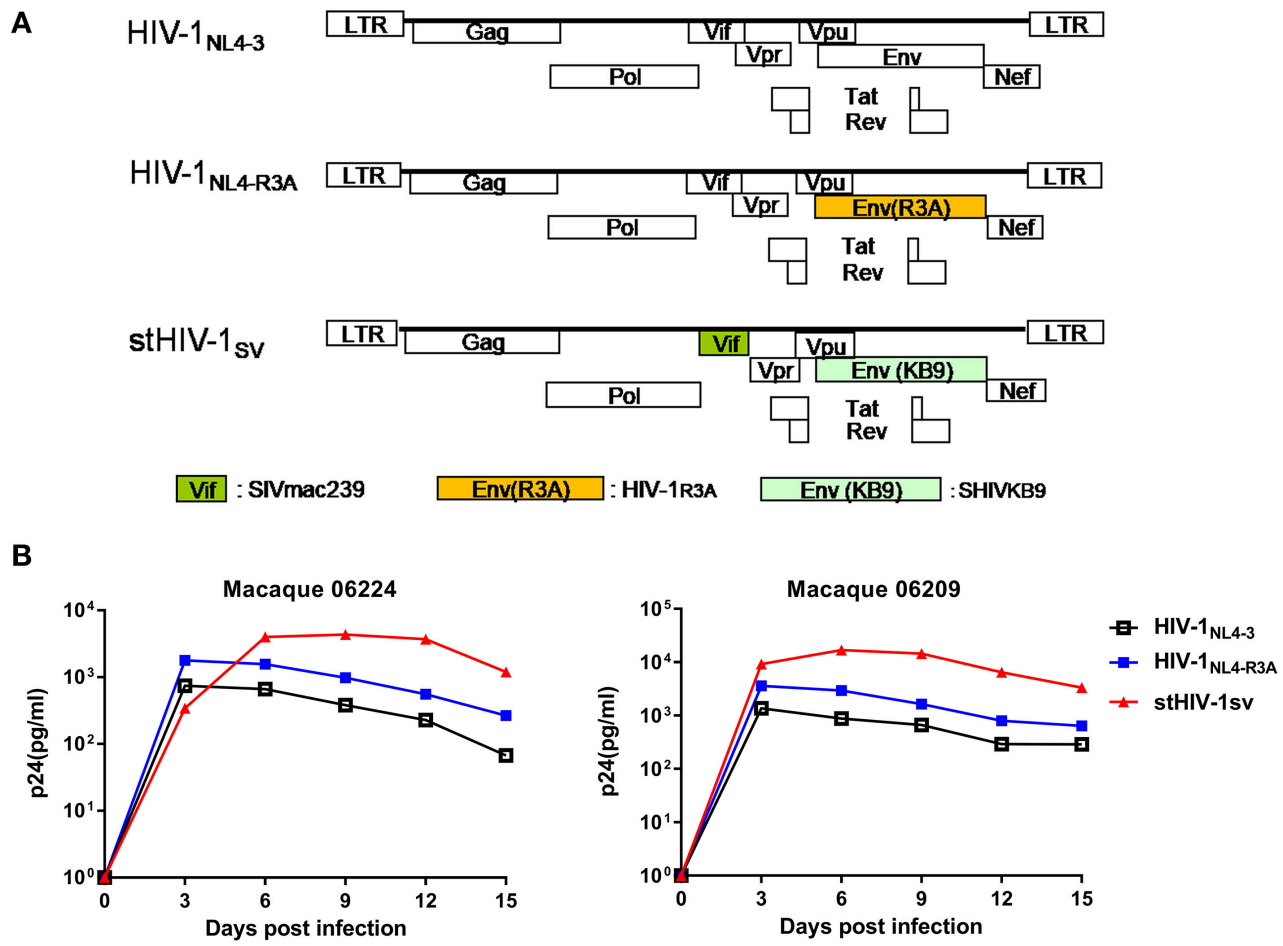
HIV-1_NL4−R3A_ and stHIV-1sv replicated more robustly than HIV-1_NL4.3_ in PBMCs of northern pig-tailed macaques. **(A)** The genomic structures of HIV-1_NL4−R3A_, and stHIV-1sv in comparison to that of HIV-1_NL4−3_. **(B)** Replication of HIV-1_NL4−3_, HIV-1_NL4−R3A_ and stHIV-1sv in PBMCs of NPMs. 1 × 10^5^ PBMCs from 2 healthy donors of NPMs were infected with these 3 viruses at an MOI of 0.01, replication was monitored by determining P24 concentration in supernatant at 3-day intervals post-infection.

In our previous study, we inoculated 4 NPMs with HIV-1_NL4−3_ and monitored the infection for about 3 years. HIV-1_NL4−3_ established successful infection in all 4 animals, with transient but persistent viremia for 6 weeks. The plasma viral load was low, peaking at 10^3^-10^4^ copies of viral gag RNA (vRNA) per ml in the first wpi, and then declining quickly (22). This implied that HIV-1_NL4.3_ was seriously inhibited *in vivo*. In this study, we generated the HIV-1_NL4−R3A_ and stHIV-1sv strains: HIV-1_NL4−R3A_ encoding a HIV-1_R3A_ *env* gene derived from HIV-1_NL4.3_ (34), and stHIV-1sv encoding a macaque-adapted HIV-1 *env* from SHIVKB9 and with *vif* gene replaced by SIVmac239 (28) (Figure 1A). Both strains encoded CCR5 and CXCR4 dual-tropic envelope proteins. When HIV-1_NL4−R3A_ and HIV-1_NL4.3_ infected PBMCs from two healthy NPM donors at a MOI of 0.01, respectively, their peak replications were achieved at day 3 suggesting poor adaptation of HIV-1 in the NPM cells. However, the p24 Gag levels increased by approximately 2.5- to 3-folds in the HIV-1_NL4−R3A_ infected cells compared to the wild type, implying switching to the R3A *env* gene improved its replication capacity in NPM cells. In contrast, stHIV-1sv carrying the SIV *vif* substitution replicated robustly in NPM cells, with increased p24 gag levels peaking on day 6 or 9, approximately 10-fold higher compared to the wild type virus, which was consistent with the observations in SPMs (28). This result suggested SIV-derived Vif protein might effectively antagonize the anti-retroviral activity of APOBEC/3F proteins in NPM cells (Figure 1B).

### HIV-1_NL4-R3A_ and stHIV-1sv can persistently replicate in northern pig-tailed macaques

**Figure 2:**
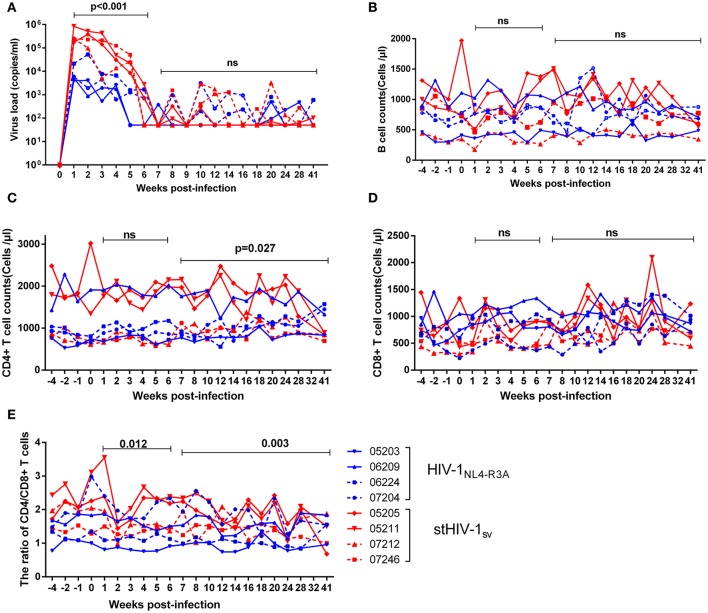
The primary infection of northern pig-tailed macaques with HIV-1_NL4−R3A_ and stHIV-1sv. 4 NPMs were infected with HIV-1_NL4−R3A_ and stHIV-1sv, respectively. **(A)** Plasma viral load, **(B)** B cell counts, **(C)** CD4+ T cell counts, **(D)** CD8+ T cell counts, **(E)** the ratio of CD4+ to CD8+ T cells in peripheral blood in the primary infection of 28 weeks were measured. *P*-value differences between HIV-1_NL4−R3A_ and stHIV-1sv infected groups were calculated by two-way analysis of variance.

To examine whether or not HIV-1_NL4−R3A_ and stHIV-1sv can infect NPM more efficiently than HIV-1_NL4−3_, 4 NPMs each were inoculated intravenously with these two viruses. The peak plasma viral loads in the NPMs infected with HIV-1_NL4−R3A_ were 10^3^-10^4^ viral RNA copies (vRNAs) per ml, with 3 NPMs showing the peak at 1 wpi and one at 2 wpi. For the NPMs infected with stHIV-1sv, the plasma viral load of 3 NPMs reached its peak (~8 × 10^5^ vRNAs copies/ml) at 1 wpi, and one showed the peak viral load (~4 × 10^5^ copies/ml) at 2 wpi. Viremia of both groups was relatively stable during the first 2 wpi, and started declining from 3 to 6 weeks. Thereafter, only occasionally virus rebounds were detected in the plasma until 41 wpi. During the first 6 weeks of primary infection, the level of plasma vRNAs of stHIV-1sv was approximately 10 fold higher than that of HIV-1_NL4−R3A_, implying that the SIV *vif* and SHIVKB9 *env* substitution improved replication capacity of HIV-1 in NPMs. However, Both HIV-1_NL4−R3A_ and stHIV-1sv could persistently produce low levels of virus particles in plasma even at late stages of the primary infection, which suggested more efficient replication than HIV-1_NL4.3_ (22), although there were still impediments to HIV-1 replication in NPMs (Figure 2A).

In addition, we examined the kinetics of the main immune cells like CD4+ and CD8+ T cells, B cells, as well as the CD4+/CD8+ ratio in peripheral blood. The peripheral CD4+ T-cell counts in the NPMs challenged with HIV-1_NL4−R3A_ and stHIV-1sv fluctuated around 1,000 and 1,500 cells/μl respectively. After 6 wpi, CD4+ T cell counts in stHIV-1sv infected NPMs presented a modest fluctuation, different from that of HIV-1_NL4−R3A_ infection (Figure 2B). Although CD8+ T-cells and B cells had little divergence in the two groups (Figures 2C,D), the CD4+/CD8+ T-cell ratio declined more rapidly in the stHIV-1sv infected NPMs compared to the HIV-1_NL4−R3A_ infected NPMs during the primary infection (Figure 2E), consistent with the respective viral loads in the two groups. This suggested greater virulence of stHIV-1sv compared to HIV-1_NL4−R3A_ in the NPMs.

### Viral DNAs and RNAs remained at relatively high levels in the PBMCs of HIV-1_NL4-R3A_ and stHIV-1sv infected NPMs

**Figure 3:**
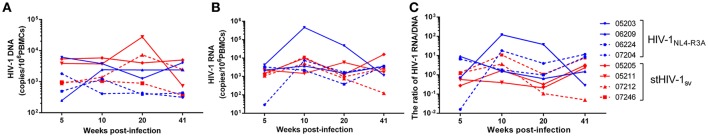
PBMC associated viral DNAs and RNAs in HIV-1_NL4−R3A_ and stHIV-1sv infected NPMs. **(A)** PBMC associated viral DNAs, **(B)** PBMC associated viral RNAs, **(C)** the proportion of viral RNAs to viral DNAs. P value differences between HIV-1_NL4−R3A_ and stHIV-1sv infected groups were calculated by two-way analysis of variance.

Since HIV-1_NL4−R3A_ and stHIV-1sv replication were greatly inhibited after 6 wpi, we assessed the numbers of viral *gag* DNA and RNA in the PBMCs of infected animals. In the absence of viremia, the level of PBMC-associated HIV-1 RNA could be considered as the indicator of HIV-1 active transcripts, and therefore a useful alternative biomarker for viral replication. PBMC-associated HIV-1 DNA on the other hand might represent a dynamic biomarker of viral persistence or viral reservoirs (34). Although viral RNA levels in the plasma decreased quickly in both HIV-1_R3A_ and stHIV-1 infected NPMs, viral DNAs and RNAs in their PBMCs remained at a relatively high level. The viral DNA was maintained at 10^3^-10^4^ per million PBMCs in both groups of infected NPMs from 5 to 41 wpi, and was slightly higher than that observed in HIV-1_NL4.3_ infection (22). Cell-associated DNA in HIV-1_NL4−R3A_ infected NPMs had a tendency of decreasing, but was not statistically significant (*p* = 0.079) (Figure 3A). Viral RNAs were highly expressed in PBMCs of HIV-1_NL4−R3A_ and stHIV-1sv infected NPMs, ranged from 10^3^ to 10^4^ per million cells from 5 to 41 wpi (Figure 3B). The levels of cell-associated HIV-1 DNAs and RNAs were comparable to the pre-therapy values observed in HIV-1 patients. However, cell free HIV-1 RNA (plasma viremia) decayed quickly and only appeared sporadically during these time points (Figure 2A). This discordance between the cell-free and cell-associated viral RNA suggested that the transcription or post-transcriptional regulation of a significant number of pro-viruses were gradually impeded, as seen with HIV-1_NL4.3_ (22). The proportion of cell-associated viral RNA relative to the DNA was higher, although not significantly (*p* = 0.29), in HIV-1_NL4−R3A_ infected NPMs (Figure 3C), which implied that more viral RNAs were inhibited to produce virus particles in HIV-1_NL4−R3A_ infection, compared to stHIV-1_sv_ infection.

### Humoral and cellular immune responses in the primary HIV-1_NL4-R3A_ and stHIV-1sv infection

**Figure 4:**
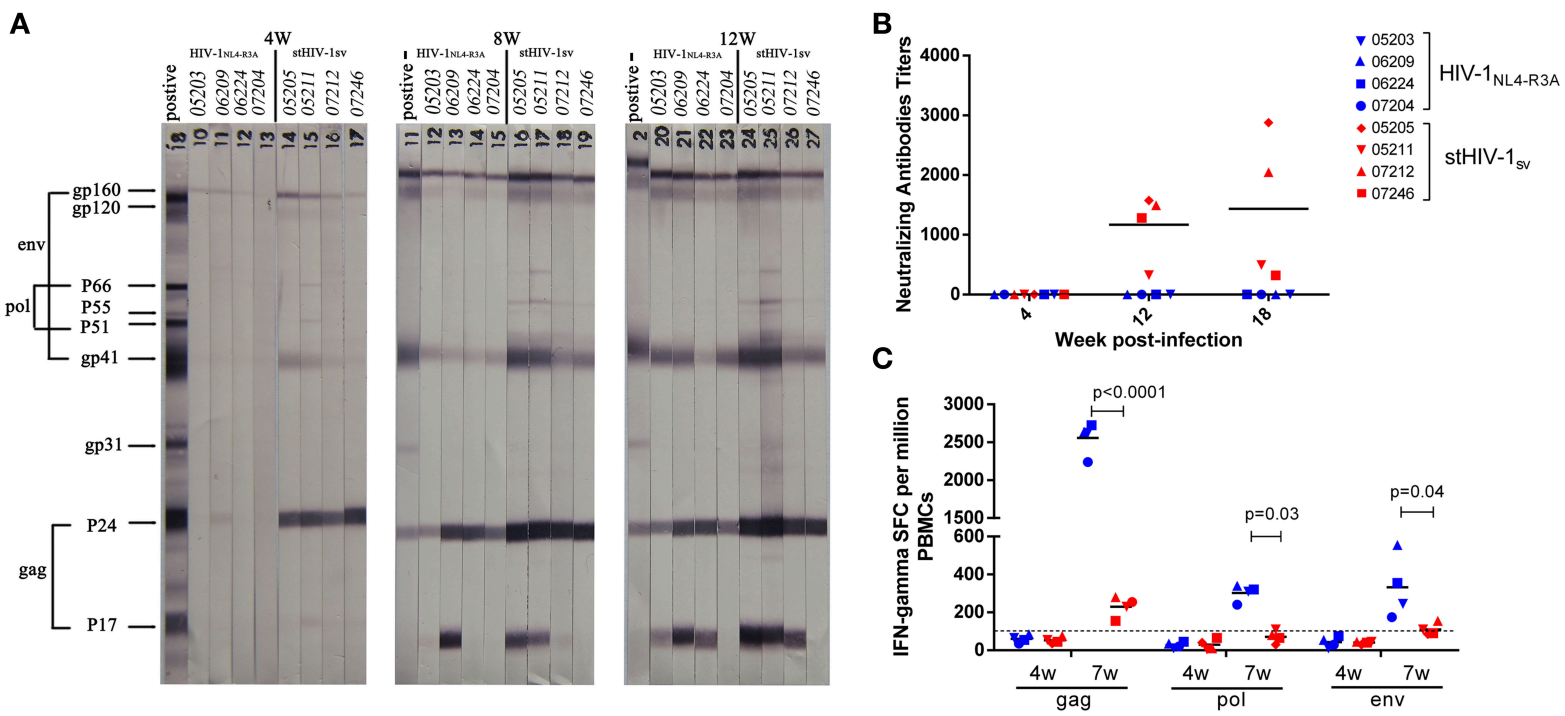
Antibody and Cotoxic lymphocyte(CTL) responses to the primary infection of northern pig-tailed macaques with HIV-1_NL4−R3A_ and stHIV-1sv. **(A)** Antibody responses to HIV-1_NL4−R3A_ and stHIV-1sv infection in NPMs were detected by western blotting analysis, the plasma at 4, 8, and 12 weeks postinfection (wpi) were used. **(B)** HIV-1 special neutralizing antibodies against these two viruses were measured respectively at 4, 12, 18 wpi. The inactivated plasma mixed with corresponding virus was added into Tzm-bl and the 50% reductions of virus infectivity (IC50) were calculated. **(C)** Cotoxic lymphocyte (CTL) responses to these two viruses were determined at 4 and 7 wpi. *P*-values were calculated by *t*-test.

In order to determine the underlying mechanism the poor reproductive status of the viruses, we evaluated the adaptive immune responses in primary infection. HIV-specific antibodies in the plasma were detected at 4, 8, and 12 wpi. At 4 wpi, only weak antibodies against HIV-1 Gag protein emerged in the stHIV-1sv infected macaques, while no antibodies against HIV-1 was generated in the HIV-1_NL4−R3A_ infected animals. The antibody levels increased dramatically after 4 wpi, particularly between 8 and 12 wpi. At 8 wpi, antibodies targeting most HIV-1 proteins were generated in HIV-1_NL4−R3A_ and stHIV-1sv infected macaques. Compared to stHIV-1sv infection, a weaker response was observed to HIV-1_NL4−R3A_. At 12 wpi, the antibody levels in both infected groups were raised and differed little, suggesting the same degree of antibody saturation in the HIV-1_NL4−R3A_ and stHIV-1sv infected macaques (Figure 4A). Interestingly, despite the rapid emergence of different HIV-specific antibodies in plasma, HIV-1_NL4−R3A_ infected macaques did not develop any detectable neutralizing antibodies against HIV-1 till 18 wpi. In contrast, the stHIV-1sv infected animals did not develop detectable neutralizing antibodies at 4 wpi but showed high levels from 12 wpi (Figure 4B). Based on the Western blotting and neutralizing antibodies assays, a more robust serological response was observed against stHIV-1sv than HIV-1_NL4−R3A_, which correlated inversely with the plasma stHIV-1sv loads. This suggested that HIV-specific antibodies, especially neutralizing antibodies, might control viral replication in the early stages of infection, particularly from 4 to 18 wpi. However, no neutralizing antibodies were detectable in HIV-1_NL4−R3A_ infected animals, implying fewer cell free virus particles that were not strong enough to induce neutralizing antibodies. In addition, other immune mechanisms prior to the production of neutralizing antibodies may also have inhibited HIV-1_NL4−R3A_ replication.

Cellular immune responses to Env, Gag, and Pol proteins were determined by IFN-γ ELISPOT assays at 4 and 7 wpi. There was no detectable HIV-specific cellular immune response in either HIV-1_NL4−R3A_ or stHIV-1sv infected macaques at 4 wpi, suggesting weakly cytotoxic T lymphocytes at the early stages of infection. At 7 wpi, both HIV-1_NL4−R3A_ and stHIV-1sv infection generated HIV-specific cellular immune responses to Env, Gag, and Pol. Unexpectedly, stHIV-1sv infected NPMs initiated only moderate but measurable CD8+ T-cell responses to HIV-1 antigens, while HIV-1 _NL4−R3A_ developed much higher level of HIV-specific cellular immune response, especially to HIV-1 Gag peptides (Figure 4C).

Taken together, there were weak adaptive immune responses at the initial stages of infection of both HIV-1 _NL4−R3A_ and stHIV-1sv in NPMs, particularly during the first 4 wpi. This explains the relatively high levels of virus replication in both groups at the initial stages. Thereafter, HIV-specific neutralizing antibodies spiked but cellular immune responses were still weak in the stHIV-1sv infected NPMs. In contrast, cellular immune responses were strong while HIV-specific neutralizing antibodies remained at baseline levels in the HIV-1 _NL4−R3A_ infected NPMs. With the emergence and enhancement of either cellular or humoral immune responses, virus replication in HIV-1_NL4−R3A_ or stHIV-1sv infected NPMs was greatly inhibited in the peripheral blood.

### Interferon-related gene expression affects HIV-1_NL4-R3A_ and stHIV-1sv replication in acute stage of infection

**Figure 5:**
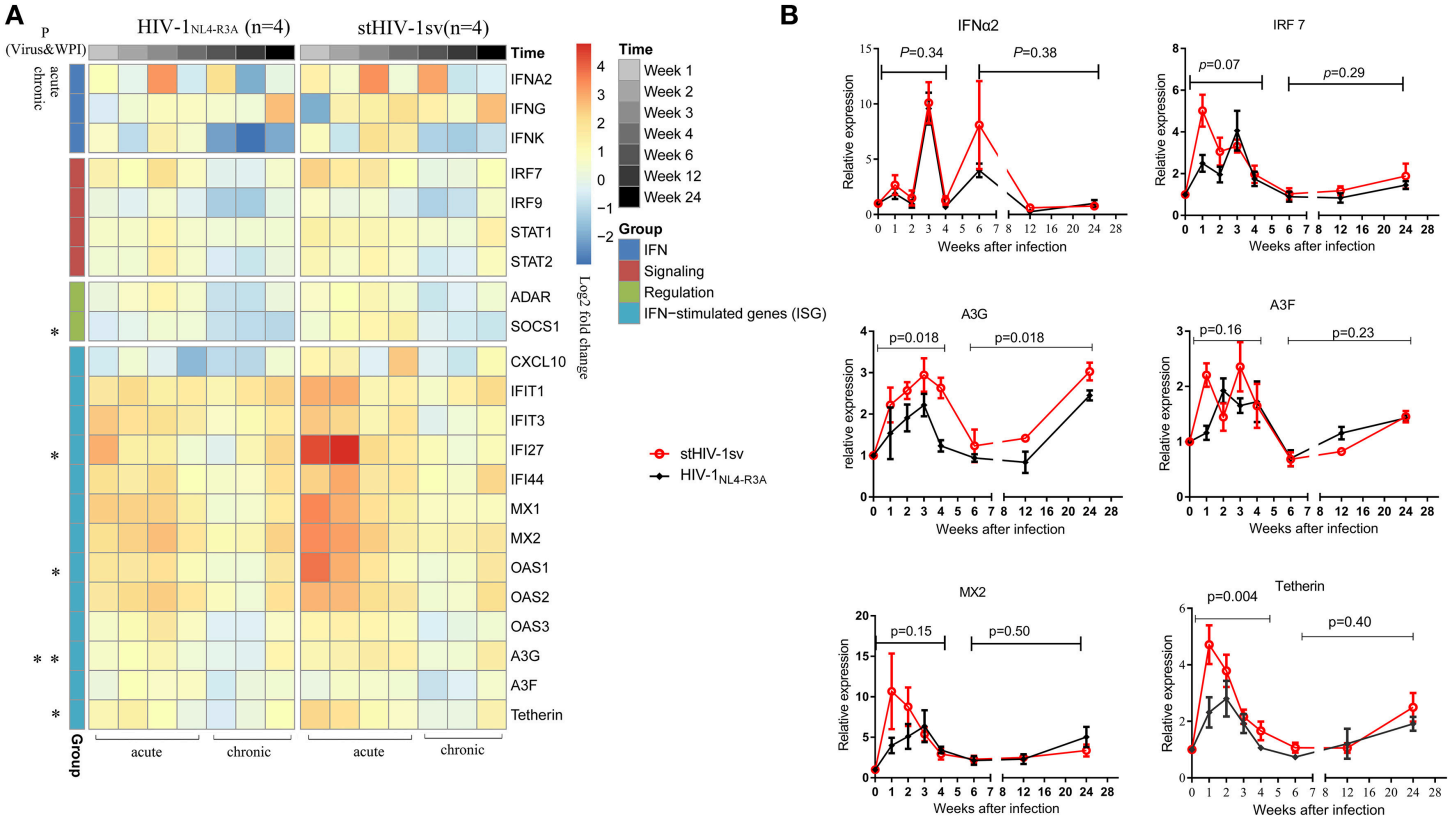
The expression of IFN-related genes during primary infection of HIV-1_NL4−R3A_ and stHIV-1sv. **(A)** The hotmap of IFN-related genes expression. 22 genes were significantly induced by HIV-1_NL4−R3A_ and stHIV-1sv infection (wpi = 1, 2, 3, 4, 6, 12, and 24) and differentially expressed in the HIV-1_NL4−R3A_ (*n* = 4) and stHIV-1sv (*n* = 4) infected macaques. Clusters were performed on the average of log2 ratios of mRNA expression relative to the basal levels before infection by the RT-PCR-based ΔΔCt method. The progressive decreases or increases in mean log2-fold-change were represented by the changes of blue to red colors. **(B)** The dynamic expression of some selected genes involved in IFNα2, IRF7, and restriction factors MX2, Tetherin, APOBEC3G and APOBEC3F. *P*-value differences between HIV-1_NL4−R3A_ and stHIV-1sv infected groups were calculated by two-way analysis of variance.

To determine the mechanism of the rapid inhibition of HIV-1 replication in the acute stage of infection, especially in the first 4 wpi when the adaptive immune responses were weak in both infected groups, the innate immune responses were examined. Using real-time PCR, we screened the RNA expression levels of some interferon-related genes, including those of interferons, interferon signaling, interferon regulation, and the interferon stimulated genes. All interferon-related genes were up-regulated, expect CXCL-10 induced by type II interferon, which decreased in the HIV-1_NL4−3A_ infected NPMs. The type I interferon stimulated genes (ISGs) were significantly up-regulated in the first 4 wpi, which could be the main reason of the rapid inhibition of HIV-1 replication in the acute stage. The two infected groups showed significantly different gene expression profiles. The stHIV-1sv infected macaques showed significantly higher expression of interferon signaling factors, especially IFI27, OAS1, APOBEC3G and Tetherin, in the first 4 wpi compared to the HIV-1_NL4−R3A_ infected animals (Figure 5A).

Restriction factors, including MX2, Tetherin, APOBEC3G, and APOBEC3F, have demonstrated to inhibit HIV-1 replication (23, 24, 35, 36). In our study, these factors peaked at 1–4 wpi, and then gradually declined to baseline levels at 6 wpi (Figure 5B). This was consistent with the changes in viral load, indicating that the host could robustly control and delay HIV-1 replication. Tetherin and APOBEC3G showed significantly higher expression levels in the stHIV-1sv infected macaques during the first 4 wpi, with APOBEC3G maintaining high levels throughout infection, indicating an important role in controlling virus replication. Taken together, interferon-related genes expression, especially those of ISGs, might affect HIV-1 replication in the acute infection stage.

### Host restriction factor APOBEC3G/F can block HIV-1 replication

APOBEC3 proteins are not only capable of suppressing retrovirus replication but also potentially facilitate viral diversity and adaption to hosts. According to the preferred mutation type system, APOBEC3G preferentially triggers GG-to-AG hypermutation, while GA-to-AA mutation is usually induced by APOBEC3F (37, 38).

**Figure 6:**
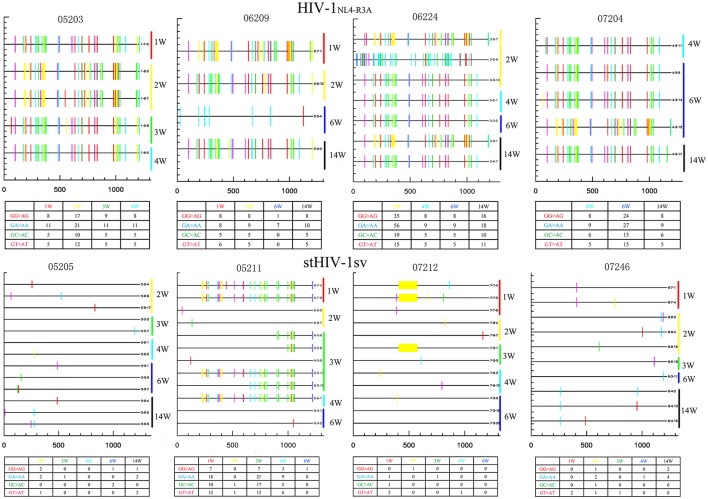
APOBEC3-induced HIV-1 hypermutation may contribute to the inhibition of virus replication during primary infection. Samples from HIV-1_NL4−R3A_ and stHIV-1sv infected 4 NPMs, respectively. The weeks post-infection were showed at the right side. Number in the table displayed the amount of hypermutation. There were more mutations caused by APOBEC3 family in HIV-1_NL4−R3A_ infected macaques than stHIV-1sv. The colors represented as: Red tick mark, GG>AG mutation (APOBEC3G pattern); Cyan tick mark, GA>AA mutation (APOBEC3F pattern); Green tick mark, GC>AC mutation; Magenta tick mark, GT>AT mutation; all other mutations; Yellow tick mark, deletion.

To determine the role of APOBEC3G and APOBEC3F in inhibiting the replication of HIV-1 and stHIV-1sv in NPMs, we amplified and sequenced the viral *env* region. There were a large number of mutations in the *env* region of HIV-1_NL4−R3A_ caused by the APOBEC3 family. The APOBEC3G/3F induced more mutations in HIV-1_NL4−R3A_ than stHIVsv, indicating its strong influence on HIV-1_NL4−R3A_ genome and its replication (Figure 6). This might be a possible explanation for the relatively low viral load and high levels of cell-associated viral RNA observed in HIV-1_NL4−R3A_ challenged macaques.

There were also many mutations in the stHIV-1sv infecting the NPM 05211, while a gene deletion was observed in the virus in animal 07212. Scanty mutations which were caused by APOBEC3 family appeared in the stHIV-1sv of NPM 05205 and 07246 (Figure 6). Therefore, SIVmac239 *vif* region substitution in stHIV-1sv might be a good antagonist for the host limiting factor APOBEC3G/3F, and thus more conducive to the virus replication. These results likely explain as to why the viral load of stHIV-1sv infected macaques was significantly higher than the HIV-1_NL4−R3A_ infected ones at the acute stage.

In addition, a large number of sequences that were consistent with the original virus were amplified in stHIV-1sv, and some amplified *env* sequences were exactly the same at different time points in all stHIV-1sv viruses. These results explain the significant inhibition of stHIV-1sv replication, despite its higher plasma viral load. The exact mechanism is still unclear, and it is likely that the expression of the other IFNs-related genes also exerted anti-viral effects, and inhibited virus replication in the acute phase.

## Discussion

We investigated the early virological, humoral and cellular immune responses, and the interferon-related gene expression profiles in NPMs following HIV-1_NL4−R3A_ and stHIV-1sv infection. NPMs can permit HIV-1_NL4−R3A_ and stHIV-1sv persistently replicate 41 weeks in the observing times of acute infection stage, with only modest fluctuations but no obvious decrease of main immune cells during the primary infection of both viruses, indicating that HIV-1_NL4−R3A_ and stHIV-1sv infection were partly inhibited by the host immune system. Therefore, it is a potential primate model of HIV-1 infection.

Adaptive immune responses likely play a key role in controlling HIV-1 infection in NPMs. The viral replication in peripheral blood decreased correspondingly with the activation of HIV-specific cytotoxic T-cells or neutralizing antibody responses. However, NPMs infected with the viruses demonstrated completely different adaptive immune response patterns: while HIV-specific cytotoxic T-cell response was strong in the HIV-1 _NL4−R3A_ infected NPMs, neutralizing antibodies were the prime immune-mediators in stHIV-1sv infected animals. In our previous studies, we have found that a long-term viral reservoir was formed in HIV-1_NL4−3_ infected NPMs, in which cell-associated pro-viral DNA and viral RNA persisted for about 3 years, although no virus particles were detectable in the peripheral blood after the first 6 wpi (22). In this study, the two novel HIV-1 viruses showed more robustly replicative potential and maintained a high level of pro-viral DNA and viral RNA in the PBMCs of NPMs at the observed time points. However, there was lower cell free viral RNA and a higher proportion of cell-associated viral RNA in HIV-1 _NL4−R3A_ infected NPMs. This might explain the induction of fewer neutralizing antibodies but higher HIV-specific cytotoxic T-cell responses after HIV-1 _NL4−R3A_ infection. This observation was agreed with that of SPMs, in which cellular immune responses were presented at 4–8 wpi and persisted to 140 wpi (14, 16), while low levels of neutralizing antibodies were observed during the observing times of 16 weeks (12).

Previous studies demonstrated that stHIV-1sv replicates more robustly than HIV-1 and can establish persistent infection in SPMs (27–30). However, stHIV-1sv did not efficiently induce degradation of APOBEC3G and APOBEC3F proteins in SPMs as well as SIV, which might result from the relatively lower expression of Vif in stHIV-1sv compared to SIV (29). In our present study, HIV-1_NL4−R3A_ and stHIV-1sv could both efficiently infect NPMs, the viral load of stHIV-1sv was significantly higher (~10–fold) than that of HIV-1_NL4−R3A_, likely resulting in a strong neutralizing antibody response. In addition, there were fewer G-to-A mutations in stHIV-1sv, implying that SIV *vif* can better antagonize the role of APOBEC3 family *in vivo*, induce degradation of APOBEC3G and APOBEC3F proteins, which is consistent with our previous study *in vitro* (39). However, there was report that APOBEC3G has the capacity to activate HIV-specific CTLs in infected T cells (40). Thus, APOBEC3G might act not only as an antiviral restriction factor, but also as an inducer of CTL response. This might be a possible reason why HIV-specific cytotoxic T-cell responses were weak in stHIV-1sv infected NPMs. This result contradicts the early reports on SPMs. In which CD8+ T-cells played a pivotal role in controlling stHIV-1sv replication and pathogenicity (28, 30). Therefore, out result might imply some differences in genetic background, as well as the immunological responses between NPMs and SPMs.

It was founded that HIV-1 or stHIV-1 could escape suppression by IFN-I in human PBMCs or CD4+ T cells, but they were highly sensitive to IFN- I in these cells of SPMs, which suggested HIV-1 might be sensitive to IFN-I induced restriction factors in non-natural hosts. Further research found the mutation in the HIV *env* region might be essential to IFN-I resistance in the cells of non-natural hosts (41, 42). In the present study, we generated two HIV-1 strains, HIV-1_NL4−R3A_ encoding an Env protein of R3A strains, while stHIV-1sv encoding a macaque-adapted Env protein from SHIVKB9, Both of them could replicate more robustly than HIV-1_NL4−3_ in the PBMCs of NPMs, suggesting HIV Env might partly contribute to escape suppression by IFN-I in PBMCs of NPMs. In addition, the role of type I IFNs and the precise time for IFN-based treatment has been widely discussed for the acute phase of retroviral infection (43). A recent study showed that both IFN signaling deficiency and increase in the first weeks of SIV infection would lead to rapid AIDS progression (44), which is paradoxical. Therefore, it is still not clear what role IFN-1 signaling might play in acute infection stage of HIV-1. In our study, we observed that early IFN-I signaling was up-regulated with the viral replication, and the levels of ISGs expression was positively correlated with the change in the load of both viruses. These results indicated a possible role of IFN-I signaling in the innate immune control of virus replication in the early stages of infection, and implied the *env* regions of HIV-1_NL4−R3A_ and stHIV-1sv were not sufficient to enhance viral replication after the IFN-I signaling was activated.

In conclusion, this study examined some immunological and virological characteristics of HIV-1_NL4−R3A_ and stHIV-1sv infected NPMs, and demonstrated that these two HIV-1 strains can persistently replicate in NPMs. The up-regulation of interferon related genes like APOBEC3G and APOBEC3F, as well as HIV-1 specific cytotoxic T-cell and neutralizing antibody responses might contribute to inhibit viral replication in the acute phase of infection. It suggests some distinctions in physiological and immunologic responses between SPMs and NPMs following HIV-1 infection. Thus, NPM has high values to develop as a novel HIV-1 animal model.

## Ethics statement

This is to confirm that the research protocol of the study entitled Research on HIV-1 infection in Northern pig-tailed macaque (Principal investigator: Y-TZ), has been reviewed and approved by the internal review board of Kunming Institute of Zoology, Chinese Academy of Sciences (approval ID:SMKX-2013026, approval date:2013/8/26).

## Author contributions

Y-TZ designed research. WP, J-HS, YL, X-LZ, H-YZ, and JJ performed research. WP and J-HS analyzed data. WP, J-HS, and Y-TZ wrote the paper.

### Conflict of interest statement

The authors declare that the research was conducted in the absence of any commercial or financial relationships that could be construed as a potential conflict of interest.
